# A systematic review protocol on small/kiddie cigarette packaging size and its impact on smoking

**DOI:** 10.1186/s13643-019-1263-6

**Published:** 2020-01-13

**Authors:** Halizah Mat Rifin, Wan Shakira Rodzlan Hasani, Miaw Yn Jane Ling, Tania Gayle Robert Lourdes, Thamil Arasu Saminathan, Nur Liana Ab Majid, Ahzairin Ahmad, Hasimah Ismail, Muhammad Fadhli Mohd Yusoff

**Affiliations:** Institute for Public Health, National Institutes of Health, Ministry of Health Malaysia, Setia Alam, Shah Alam, Selangor Malaysia

**Keywords:** Smoking, Kiddie packs, Small packs, Mini packs, Cigarette, Packaging, Size

## Abstract

**Background:**

Small/kiddie cigarette packs consist of less than 20 cigarette sticks. Kiddie packs were recently proposed to be reintroduced by the tobacco industry with an excuse to prevent consumers from buying illicit cigarettes. By reintroducing kiddie packs, cigarettes will inevitably be more affordable and this would appeal to lower-income consumers especially teens. In this systematic review, we aimed to identify the impact of kiddie packs on smoking, specifically on smoking initiation, the urge/tendency to buy cigarettes and attempts to reduce cigarette consumption.

**Methods:**

This systematic review will be based on the review of original articles on the impact of kiddie packs on smoking. There is no restriction on the publication dates. The Cochrane Central Register of Controlled Trials, PubMed, EMBASE, Web of Science and Scopus will be searched to retrieve potential original articles. Additional records identified through other sources: Google Scholar, as well as Journal of Substance Use and Tobacco Control, are also to be searched. These will include original articles in any language which included all study designs (randomised controlled trials, quasi experimental and experimental studies, observational cross-sectional and cohort studies) comparing kiddie packs with regular cigarette packs. The primary outcomes of interest will be initiation of smoking and urge/tendency to buy cigarettes in the general population and attempts to reduce cigarette consumption among current smokers. Secondary outcomes will be the prevalence of smoking using kiddie packs among the current smokers.

**Discussion:**

This systematic review will provide evidence to support the impact of kiddie packs on smoking in terms of smoking initiation, smoking prevalence, urge/tendency to purchase cigarettes and attempts to reduce cigarette consumption. The findings from this review could be helpful to policymakers in regulating kiddie packs to control the consumption of tobacco.

**Systematic review registration:**

PROSPERO CRD42018102325

## Background

Globally, in 2015, over 1.1 billion people smoked tobacco [1]. Tobacco use whether smoked (manufactured cigarette, kretek and hand-rolled cigarette) or smokeless (snuff tobacco, electronic cigarette and chew tobacco) was responsible for the mortality (mostly premature) of about six million people per year according to the World Health Organization (WHO) [2]. Cigarettes have been sold in tins, cartons, packs, small/kiddie packs and as loose sticks [3]. Small/kiddie cigarette packaging (hereafter referred to as kiddie packs) consists of less than 20 cigarettes [4]. It has been sold in packages of 15, 10 or 5 cigarettes in many countries [5]. Among the countries where kiddie packs are available include Indonesia, Thailand and Philippines [6].

According to the British American Tobacco (2004) [7], smaller packs might encourage underage smoking but a pack of 10s might support moderation and encourage quit smoking among heavy smokers. However, it can also encourage the low-income consumers, mainly teens and minors to purchase kiddie packs as prices plunge with quantity reduction [3]. Studies have shown that tobacco consumption dropped in response to higher prices [8–10]. Based on 2014 Global Youth Tobacco Survey, three out of five Indonesian students aged between 13 and 15 could buy cigarettes easily probably due to the availability of single sticks [11]. This was supported by another study conducted in Bali, where more than half of retailers in Denpasar sold cigarettes, in single sticks to young people as it is affordable and more accessible [12].

Based on the above stated debates, the WHO Framework Convention on Tobacco Control (FCTC) recommends countries to eliminate sale of kiddie packs and single sticks. In addition, Article 16 the WHO’s framework Convention on Tobacco Control (FCTC) [13] states that comprehensive policies and effective enforcement strategies are recommended in order to stop the sale of single stick cigarettes and kiddie packs. In 2012, 84 countries (of the FCTC) had policies to prevent the sales of single sticks or kiddie packs [14].

The government of South Australia was the first in the world to establish a ban on kiddie packs in 1986 [15] followed by Canada in 1994 [16]. In Asia, Singapore (2002), Brunei (2005), Laos (2009), Malaysia (2010), Cambodia (2015) and Vietnam (2016) had banned kiddie packs from the market to prevent teens from smoking [17]. Despite the ban on kiddie packs in some countries, the tobacco industry is trying to reintroduce kiddie packs with the excuse of combatting the surge of contraband cigarettes [18].

There is a need to expand the scope of such findings to arrive at an evidence-based conclusion. To our knowledge, there is no published systematic review that addresses our questions, which are as follows: (1) What is the impact of kiddie packs on initiation of smoking in the general population? (2) What is the impact of kiddie packs on urge/tendency to buy cigarette in the general population? (3) What is the impact of kiddie packs on attempt to reduce cigarette consumption among current smokers? (4) What is the prevalence of smoking kiddie packs among current smokers? We have addressed our objectives through a comprehensive protocol targeting all studies (randomised controlled trials, quasi experimental and experimental studies, observational cross sectional and cohort studies) in this area from all years, in order to identify the impacts of kiddie packs on the initiation of smoking and urge/tendency to buy cigarettes in the general population, and attempt to reduce cigarette consumption and prevalence of smoking kiddie packs among current smokers.

## Methods/design

### Research objective

The aim of this review is to identify the effects of kiddie packs on smoking as compared to regular cigarette packaging. The objectives are:

1. To identify the impact of kiddie packs specifically on the initiation of smoking, urge/tendency to buy cigarettes in the general population
2. To identify the impact of kiddie packs on attempt to reduce cigarette consumption among current smokers
3. To determine the prevalence of smoking using kiddie packs among current smokers

### Inclusion/exclusion criteria

#### Study design

We will follow the Preferred Reporting Items for Systematic review and Meta-Analyses (PRISMA) guidelines and its extension for protocols (PRISMA-P) [19] [see Additional file 1: PRISMA-P checklist]. Studies written in any language and all original articles published without restriction on the publication dates will be included in this review. Original articles of any level of rigour including quantitative and qualitative studies will be considered. The rigour of the study is determined according to the research design and method, reliability, validity, and openness and transparency of the study.

We plan to include all studies (randomised controlled trials, cluster randomised controlled trial, quasi experimental and other experimental studies and observational studies) in this area from their earliest record. We will exclude guidelines, conference papers, commentaries, editorial or opinion pieces. Publications focused on the use of cigarette packs other than kiddie packs will be excluded.

In order to evaluate the tobacco industry influence on the study, we will look into full paper and check for sponsorship and conflict of interest. If the research is being sponsored by a tobacco company, we will consider bias on the study and will state in a specific column that the research is sponsored by a tobacco company.

#### Population

We will include the public at large on initiation of smoking of kiddie packs and have the urge/tendency to buy kiddie packs. For attempts to reduce cigarette consumption, we will only include current smokers.

#### Exposure

We will include studies that specifically aim to evaluate the impact of kiddie packs

#### Comparators

The impact of kiddie packs will be compared to regular sized cigarette packs.

#### Outcomes

The primary outcomes of interest will be (1) initiation of smoking in the general population, (2) urge/tendency to buy kiddie packs in the general population and (3) attempts to reduce cigarette consumption among current smokers. Secondary outcome will be the prevalence of smoking using kiddie packs among current smokers.

#### Operational definition

Initiation of smoking was defined as when an individual first smoked a whole cigarette [20]. It is also meant changing one’s status from a non-smoker to a smoker [21]. Urge/tendency to buy is defined as a sudden, powerful and persistent urge to buy something which is immediately experienced by a consumer. Cigarette package size in a combination with retail displays was a contributor to impulse sales based on circulated review of advertising in a Canadian retailer trade press magazine (Your Convenience Manager) [4]. Smoking urge will be measured using a validated scale such as ten-item Questionnaire of Smoking Urge (QSU-brief) [22] or any scale as stated by the authors in the included studies. The validated scale is used to measure the craving for cigarettes, as thus in this study, it will measure the urge/tendency to buy kiddie packs. Reduced cigarette consumption is defined reducing the number of cigarettes smoked each day. Reducing the number of cigarettes is a common strategy used by smokers to move towards smoking cessation, to reduce harm or for saving money [23].

#### Search strategy

The search strategy aims to find published articles and will include a three-stage protocol: (1) An initial limited search of PubMed and Cochrane Library of Systematic Review will be undertaken; this will be followed by analysis of the text words found in the titles and abstracts, as well as index terms used to describe each article, these will be used to build a keyword list. Literature search strategy will include combinations of Medical Subject Headings (MeSH) and text words related to “cigarette” and “mini pack” OR “kiddie packs” OR “small packs” and “Smoking initiation” OR “impulse to buy” OR “urge to buy” OR “tendency to buy” OR “smoking reduction”. No restriction on publication date will be applied. (2) For the second search, all identified keywords and index terms will be used, then it will be undertaken across all included databases. Databases to be included are PubMed, Cochrane Library of Systematic Review, Embase, Web of Science and Scopus. Additional records identified through other sources: Google Scholar, as well as Journal of Substance Use and Tobacco Control, are also to be searched. Studies will be in any languages and there is no restriction on the publication date. (3) In the third step, the reference lists of key articles will be searched for additional studies. All studies will be considered. Selection of studies will follow PRISMA guidelines. Only those studies that met the required criteria in addition to an evaluation of allocation concealment and proper data analysis will be included in the data extraction phase. Please refer to Additional file 2: Table S1.

#### Study selection

All selected publications found in various electronic databases through the above mentioned strategies will be uploaded in Mendeley library, and duplicate records will be deleted. Titles and/or abstracts of the original publications will be screened for duplication before being assigned to two pairs of reviewers. The two pairs of reviewers will independently screen the title abstract to exclude publications that does not meet the eligibility criteria. We will exclude guidelines, conference papers, commentaries, editorials or opinion pieces. Any disagreement that arises between pairs of reviewers will be resolved through discussion among all authors.

#### Data extraction and management

Two pairs of reviewers will independently extract data from the full texts of papers that were published previously. A form will be designed accordingly (Additional file 3: Table S2). Data will be collected as follows: study, study type, sample size, exposure, population, outcome, meta-analysis finding or 16-item quality assessment tool (QATSDD) score [24] or ROBINS-I [25] or RoB 2 [26] and sponsor status. The next independent author will review the collected data. In case of disagreement, the next independent author will help to make a final decision. The data extracted will include all details specific to the review question and which fulfils the requirements for the synthesis of outcomes. We will also contact corresponding authors for key information when data are ambiguous or missing from the published study. Data extraction will be cross-checked independently.

#### Quality and bias assessment

The two pairs of reviewers will independently check each selected articles to minimise bias and also assess the study quality independently. For randomised controlled trials (RCT), we will assess the risk of bias according to the following domains as stated in the Cochrane Handbook for Systematic Review of Intervention (Cochrane risk-of-bias tool for randomised trials) [27], which include random sequence generation, allocation concealment, blinding of outcome assessment, incomplete outcome data, selective outcome reporting and other bias. RoB 2 is a revised version of Cochrane tool which focuses on different aspects of trial design, conduct and reporting. It is structured into fixed set of domain of bias. A series of questions that elicit information relevant to risk of bias are aimed within each domain [26].

We will not consider the domain of “Blinding of participants and personnel” because this is impossible in all trials evaluating kiddie packs.

We will grade the risk of bias for each domain as high, low or unclear and provide information from the study report together with a justification for our judgement in the “Risk of bias” tables as stated in the Cochrane Handbook of Systematic Reviews of Interventions. We will classify the included studies to be at high, low or unclear risk of bias in each of these domains. We will consider the study as having low risk of bias if the methods employed in the study were sufficient to enable a reliable interpretation of the results with regard to outcome measures. We will consider a study as high risk of bias if the methods employed raise doubt on the reliability of its effectiveness. We will judge the study as having an unclear risk of bias if there is no adequate information provided, or if the risk of bias of the method employed is unknown.

We will present the results of the risk of bias analysis in tables and figures (Additional file 4: Table S3). For included studies other than RCTs, bias and study quality assessment will be based on the 16-item quality assessment tool (QATSDD) [24] (Additional file 5: Table S4) or Risk Of Bias In Non-Randomised Studies - of Interventions (ROBINS-I) [25].

QATSDD is a validated quality assessment designed for a heterogeneous study. The tool consists of 16 criteria each with a score ranging between 0 and 3, with 3 being the best. A score of 0 is awarded if authors have not included the level of detail required to make a judgement for a quality criterion. For each paper, the scores will be added and will be divided by the maximum possible score to report the paper’s overall quality score. The maximum score for mixed papers is 48 and 42 for qualitative or quantitative. Then, it will be converted into a percentage. Bias assessment criteria include identification of selection bias, information bias or confounding. Results of non-randomised studies that compare health effects of two or more interventions/exposures will be assessed using ROBINS-I [25].

#### Analysis

If we can find multiple studies that provide usable data in any single comparison, we will perform a meta-analysis [28]. If studies are statistically heterogeneous, we will use a random-effects model; otherwise, we will use a fixed-effect model. When we use the random-effects model, we will conduct a sensitivity check by using the fixed-effect model to reveal differences in results. We will include a 95% confidence interval (CI) for all estimates. We will have described skewed data reported as medians and interquartile range. If multiple trial arms are reported in a single trial, we will include only the relevant arms. We will compare the same trial arms in the same meta-analysis, to avoid double-counting. We will describe the included studies using forest plot and table.

We will conduct a meta-analysis if we can pool a minimum of two similar studies with the same category of population, exposure, comparison and outcome. We will describe the included studies using forest plot and table. If there are studies with distinctly different characteristics, for example different categories of exposure, the data will be separated into subgroups and the total pool estimate will not be provided. The data analysis will follow the strategies in the Cochrane Handbook for Systematic Reviews of Intervention for data management [29]. The data will be analysed using the intention-to-treat principle, i.e. using the original numbers of randomised participants allocated in the study arm as our denominator. We will pool all included studies with the information on prevalence and will produce a pool prevalence analysis using Stata Statistical Software: Release 12 [30]. We will use a random effect model to compute the pool prevalence. Heterogeneity will be determined by examining the population as similar as when they exposed and produce outcome at similar time or duration such as 2 months, 1 year or more than 10 years. Then, it will pool the studies together using the forest plot and quantify the impact using *I*^2^ statistic. More than 75% will be considered as heterogeneous. If formal meta-analysis is not possible due to the heterogeneous nature of studies settings, designs and outcome measures, studies with similar theme components will be grouped together for narrative synthesis. The extracted data will be summarised in a table and a narrative review will be prepared.

#### Descriptive analysis

A narrative synthesis of the outcomes of the selected studies will be presented in the final review. This will include the following:

1. The impact of kiddie cigarette packs on smoking: in terms of initiation of smoking, urge/tendency to buy the cigarettes and attempt to reduce cigarette consumption.
2. Targeted population and its characteristics: age or other sociodemographic characteristics
3. Exposure outcome: prevalence of smoking, age group that initiates smoking, and whether the kiddie packs increase initiation of smoking and increase the urge/tendency to buy the cigarette or attempt to reduce cigarette consumption.
4. The reason why kiddie packs could initiate smoking, lead the urge/tendency to buy cigarettes and attempt to reduce cigarette consumption.

## Discussion

This systematic review will provide evidence to support the impact of kiddie packs on smoking in terms of smoking initiation, smoking prevalence and the urge/tendency to buy cigarettes and attempt to reduce cigarette consumption. Based on the findings, this will help the policymakers to regulate kiddie packs as an effective tobacco control initiative. Each party needs to protect the public health policies on tobacco control from commercial and other tobacco industry interests based on the national law of the country.

## Supplementary information

**Additional file 1.** PRISMA-P checklist.

**Additional file 2: Table S1.** Key search strategy.

**Additional file 3: Table S2.** Data extraction sheet.

**Additional file 4: Table S3.** Study/risk of bias for individual included Randomised Controlled Trial studies.

**Additional file 5: Table S4.** Quality Assessment Checklist.

## Data Availability

Not applicable

## Abbreviations

PROSPERO: International Prospective Register of Systematic Reviews
WHO: World Health Organization
FCTC: Framework Convention on Tobacco Control
QATSDD: 16-item quality assessment tool
PRISMA: Preferred Reporting Items for Systematic review and Meta-Analyses
PRISMA-P: Preferred Reporting Items for Systematic review and Meta-Analyses guidelines and its extension for protocols
ROBINS-I: Risk Of Bias In Non-randomised Studies - of Interventions
RoB 2: The Revised Cochrane Risk of bias tool for randomized trials

## References

[1] World Health Organization. Global Health Observatory (GHO) data: Tobacco control: Prevalence of tobacco smoking. http://www.who.int/gho/tobacco/use/en/ . Accessed 5 Nov 2018

[2] World Health Organization. Tobacco Free Initiative (TFI): WHO global report on trends in tobacco smoking 2000-2025- First edition. https://www.who.int/tobacco/publications/surveillance/reportontrendstobaccosmoking/en/ . Accessed 3 July 2019

[3] Singh MDV, Kumar R, Kumar AM (2017). ‘Loose’ cigarettes association with intensity of smoking: A secondary data analysis from Global Adult Tobacco Survey, India 2009-10. J Sci Soc.

[4] Dewhirst T (2017). Package size matters: tobacco packaging, retail merchandising and its influence on trial and impulse sales Tobacco Control. Tob Control.

[5] U.S. Department of Health and Human Services (2000). Reducing Tobacco Use: A Report of the Surgeon General.

[6] Southeast Asia Tobacco Control Alliance (2019). A snapshot of the tobacco industry in ASEAN Region.

[7] The sensible regulation of Tobacco-British American Tobacco’s views. British American Tobacco; 2000. http://legacy.library.ucsf.edu/tid/zye51a99/pdf Accessed 3 Dec 2018

[8] World Health Organization (2008). WHO Report on the Global Tobacco Epidemic. MPOWER package.

[9] Chaloupka FJ, Warner KE (2000). The economics of smoking. Handb Health Econ.

[10] Jha P, Chaloupka FJ. Tobacco control in developing countries: Oxford University Press; 2000.

[11] World Health Organization, Regional Office for South-East Asia (2015). Global Youth Tobacco Survey (GYTS): Indonesia report, 2014.

[12] Astuti PAS, Freeman B. Protecting young Indonesian hearts from tobacco. The Conversation https://theconversation.com/protecting-young-indonesian-hearts-from-tobacco-97554 . Accessed 19 Aug 2019.

[13] World Health Organization (2003). WHO Framework Convention on Tobacco Control.

[14] Lal PKR, Ray S, Sharma N, Bhattarcharya B, Mishra D, Sinha MK, Chistian A, Rathinam A, Singh G (2015). The single cigarette economy in India- a Back of the Envelope Survey to Estimate its Magnitude. Asian Pac J Cancer Prev.

[15] Chapman S, Byrne F, Carter SM. “Australia is one of the darkest markets in the world”: the global importance of Australian tobacco control. Tobacco Control 2003;12:iii1-iii3. 10.1136/tc.12.suppl_3.iii1 PMC1766118 14645941

[16] Cunningham R. Smoke and Mirrors, The Canadian Tobacco War: International Development Research Centre; 1996.

[17] Tan YL, Dorotheo U (2016). The Tobacco Control Atlas: ASEAN Region.

[18] No to kiddie pack cigarettes - Dr Subramaniam. Bernama http://www.bernama.com/en/general/news.php?id=1428438%0A%0A Accessed 5 July 2018.

[19] Moher D, Shamseer L, Clarke M, Ghersi D, Liberati A, Petticrew M, Shekelle P, Stewart LA. Preferred reporting items for systematic review and meta-analysis protocols (PRISMA-P) 2015 statement. Syst Rev. 2015;4(1). 10.1186/2046-4053-4-1 PMC4320440 25554246

[20] Von Ah D, Ebert S, Ngamvitroj A, Park N, Kang DH (2005). Factors related to cigarette smoking initiation and use among college students. Tob Induc Dis.

[21] Nonnemaker JM, Farrelly MC (2011). Smoking initiation among youth: the role of cigarette excise taxes and prices by race/ethnicity and gender. J Health Econ.

[22] West R, Ussher M (2010). Is the ten-item Questionnaire of Smoking Urges (QSU-brief) more sensitive to abstinence than shorter craving measures?. Psychopharmacology (Berl).

[23] McNeill A (2004). Harm reduction. BMJ.

[24] Sirriyeh R, Lawton R, Gardner P (2012). Reviewing studies with diverse designs: the development and evaluation of a new tool. J Eval Clin Pract.

[25] Cochrane Methods Bias: ROBINS-I. Cochrane https://methods.cochrane.org/bias/risk-bias-non-randomised-studies-exposures . Accessed 3 July 2019

[26] RoB 2: A revised Cochrane risk-of-bias tool for randomised trials. Cochrane https://methods.cochrane.org/bias/resources/rob-2-revised-cochrane-risk-bias-tool-randomised-trials . Accessed 1 July 2019

[27] Higgins JPT, Savović J, Page MJ, Elbers RG, Sterne JAC. Chapter 8: Assessing risk of bias in a randomized trial. In: Higgins JPT, Thomas J, Chandler J, Cumpston M, Li T, Page MJ, Welch VA, editors. Cochrane Handbook for Systematic Reviews of Interventions version 6.0 (updated July 2019): Cochrane; 2019. Available from www.training.cochrane.org/handbook Accesed 1 Aug 2019.

[28] Egger M, Smith GD (1998). Bias in location and selection of studies. BMJ.

[29] Higgins JPT, Thomas J, Chandler J, Cumpston M, Li T, Page MJ, Welch VA (editors). Cochrane Handbook for Systematic Reviews of Interventions version 6.0 (updated July 2019). Cochrane, 2019. Available from www.training.cochrane.org/handbook. 10.1002/14651858.ED000142 PMC10284251 31643080

[30] StataCorp (2011). Stata Statistical Software: Release 12.

