# Supplementary material for: A systematic review protocol on small/kiddie cigarette packaging size and its impact on smoking
Source: Syst Rev. 2020 Jan 13;9:13. doi: 10.1186/s13643-019-1263-6 (PMC6958659; doi:10.1186/s13643-019-1263-6)
Supplement: Supplementary file 2 — Additional file 2: Table S1. Key search strategy. [file 13643_2019_1263_MOESM2_ESM.docx]

**Table S1:Key search strategy**

| **Criteria 1** |  | **Criteria 2** |  | **Criteria 3** |
| --- | --- | --- | --- | --- |
| “Manufactured cigarette” | AND | Pack* | AND | “Smoking initiation” |
| “Manufactured cigarettes” |  | kiddie |  | “Urge to buy” |
| Smoking |  | “Small packet” |  | “Tendency to buy” |
| Smoke |  | “Small packets” |  | “impulse to buy” |
| Tobacco |  | “Small package” |  | "smoking reduction" |
| Cigarette |  | “Small packages” |  | “intend” |
| Cigarettes |  | “Kiddie pack” |  | “desire” |
|  |  | “Kiddie packs” |  | “consumption” |
|  |  | “Kiddie packet” |  |  |
|  |  | “Kiddie packets” |  |  |
|  |  | “Kiddie package” |  |  |
|  |  | “Kiddie packages: |  |  |
|  |  | “Small box” |  |  |
|  |  | “Kiddie box” |  |  |
|  |  |  |  |  |

**1#:First key search strategy (Criteria 1)**

( “Manufactured cigarette” OR “Manufactured cigarettes” OR smoking OR Smoke OR tobacco OR cigarette OR cigarettes)

**2#:Second key search strategy (Criteria 2)**(pack* OR kiddie OR “mini” OR “Small packet” OR “Small packets” OR “Small package” OR “Small packages” OR “Kiddie pack” OR “Kiddie packs” Or “Kiddie packet” OR “Kiddie packets” OR “Kiddie package” OR “Kiddie packages” OR “Small box” OR “Kiddie box” OR “mini pack”)

**#3: Third key search strategy (Criteria 3)**

(“Smoking initiation” OR "impulse to buy" OR "urge to buy" OR "tendency to buy” OR "smoking reduction" OR “intend” OR “desire” OR “consumption”)
