# Supplementary material for: A systematic review protocol on small/kiddie cigarette packaging size and its impact on smoking
Source: Syst Rev. 2020 Jan 13;9:13. doi: 10.1186/s13643-019-1263-6 (PMC6958659; doi:10.1186/s13643-019-1263-6)
Supplement: Supplementary file 3 — Additional file 3: Table S2. Data extraction sheet. [file 13643_2019_1263_MOESM3_ESM.docx]

| **Study** | **Study type** | **Sample size** | **Exposure**  **(Size of packaging)** | **Population** | **Outcome**  **(Data collection and analysis)** | **Meta analysis finding/QAT Score/ ROBIN-I /RoB2** | **Sponsor status**  **(sponsored by a tobacco company/ researcher initiation)** |
| --- | --- | --- | --- | --- | --- | --- | --- |
|  |  |  |  |  |  |  |  |
|  |  |  |  |  |  |  |  |
