# Supplementary material for: A systematic review protocol on small/kiddie cigarette packaging size and its impact on smoking
Source: Syst Rev. 2020 Jan 13;9:13. doi: 10.1186/s13643-019-1263-6 (PMC6958659; doi:10.1186/s13643-019-1263-6)
Supplement: Supplementary file 4 — Additional file 4: Table S3. Study/risk of bias for individual included Randomised Controlled Trial studies. [file 13643_2019_1263_MOESM4_ESM.docx]

| **Study** | **Random sequence generation** | **Allocation concealment** | **Blinding of outcome assessment** | **Incomplete outcome data** | **Selective outcome reporting** | **Other bias** | **Overall** |
| --- | --- | --- | --- | --- | --- | --- | --- |
|  |  |  |  |  |  |  |  |
|  |  |  |  |  |  |  |  |
