# Supplementary material for: A systematic review protocol on small/kiddie cigarette packaging size and its impact on smoking
Source: Syst Rev. 2020 Jan 13;9:13. doi: 10.1186/s13643-019-1263-6 (PMC6958659; doi:10.1186/s13643-019-1263-6)
Supplement: Supplementary file 5 — Additional file 5: Table S4. Quality Assessment Checklist. [file 13643_2019_1263_MOESM5_ESM.doc]

|  | **Score (0-3)** | | | |
| --- | --- | --- | --- | --- |
| **Criteria** | Paper: | Paper: | Paper: | Paper: |
| 1. Explicit theoretical framework |  |  |  |  |
| 1. Statement of aims/objectives in main body of report |  |  |  |  |
| 1. Clear description of research setting |  |  |  |  |
| 1. Evidence of sample size considered in terms of analysis |  |  |  |  |
| 1. Representative sample of target group of a reasonable size |  |  |  |  |
| 1. Description of procedure for data collection |  |  |  |  |
| 1. Rationale for choice of data collection tool(s) |  |  |  |  |
| 1. Detailed recruitment data |  |  |  |  |
| 1. Statistical assessment of reliability and validity of measurement tool(s)   (Quantitative only) |  |  |  |  |
| 1. Fit between stated research question and method of data collection   (Quantitative only) |  |  |  |  |
| 1. Fit between stated research question and format and content of data collection tool e.g. interview schedule   (Qualitative only) |  |  |  |  |
| 1. Fit between research question and method of analysis |  |  |  |  |
| 1. Good justification for analytic method selected |  |  |  |  |
| 1. Assessment of reliability of analytic process   (Qualitative only) |  |  |  |  |
| 1. Evidence of user involvement in design |  |  |  |  |
| 1. Strengths and limitations critically discussed |  |  |  |  |
| **Score total/maximum score possible** |  |  |  |  |
